# Prevention of tumour cell apoptosis associated with sustained protein kinase B phosphorylation is more sensitive to regulation by insulin signalling than stimulation of proliferation and extracellular signal-regulated kinase

**DOI:** 10.1007/s11010-017-2996-y

**Published:** 2017-03-18

**Authors:** Christoph Schmid, Claudia Ghirlanda, Markus Niessen

**Affiliations:** 1 Division of Endocrinology, Diabetology and Clinical Nutrition, University Hospital of Zurich, Raemistrasse 100, 8091 Zurich, Switzerland; 2 Competence Centre for Systems Physiology and Metabolic Diseases, Swiss Federal Institute of Technology (ETH) Zurich, 8093 Zurich, Switzerland

**Keywords:** Osteosarcoma cells, IGF1, Insulin, Apoptosis, PI3K, Obesity

## Abstract

Insulin controls blood glucose while insulin-like growth factor (IGF) 1 is an important growth factor. Interestingly, both hormones have overlapping bioactivities and can activate the same intracellular signal transduction cascades. Growth control (mainly by IGF1) and metabolic function (predominantly by insulin) are believed to depend on activation of extracellular signal-regulated kinases (ERKs) 1/2 and protein kinase B (Akt/PKB), respectively. Therefore, insulin analogues that are used to normalize blood glucose are tested for their ability to preferentially activate Akt/PKB but not ERK1/2 and mitogenesis. Growth hormone, IGF1, and hyperinsulinemia are associated with increased risk of growth progression of some cancer types. To test if continuous exposure to insulin can favour tumour growth, we studied insulin/IGF1-dependent activation of ERK1/2 and Akt/PKB by Western blotting, inhibition of apoptosis by ELISA, and induction of proliferation by [^3^H]-thymidine incorporation in Saos-2/B10 osteosarcoma cells. IGF1 and insulin both induced proliferation and prevented apoptosis effectively. Regulation of apoptosis was far more sensitive than regulation of proliferation. IGF1 and insulin activated PKB (Akt/PKB) rapidly and consistently maintained its phosphorylation. Activation of ERK1/2 was only observed in response to IGF1. Loss of p-Akt/PKB (but not of p-ERK1/2) was associated with increased apoptosis, and protection from apoptosis was lost when activation of Akt/PKB was inhibited. These findings in Saos-2/B10 cells were also replicated in the A549 cell line, originally derived from a human lung carcinoma. Therefore, IGF1 and insulin more likely (at lower concentrations) enhance tumour cell survival than proliferation, via activation and maintenance of phosphatidylinositol 3-kinase activity and p-Akt/PKB.

**Electronic supplementary material:**

The online version of this article (doi:10.1007/s11010-017-2996-y) contains supplementary material, which is available to authorized users.

## Introduction

Sufficient fuel and nutrient supply and growth hormone (GH) action are important for normal growth; in vivo, insulin and insulin-like growth factor (IGF) 1 are intrinsically linked to anabolic, growth-friendly conditions. Obesity and acromegaly are deleterious conditions, associated with high insulin and IGF1 levels, and decreased life expectancy. Obesity is characterized by increased fat mass, insulin resistance, hyperinsulinemia, and an increased risk not only for type 2 diabetes but also for cancer; acromegaly is also characterized by insulin resistance but with decreased fat mass, and an excess of GH, insulin, and IGF1. Although the presence of anabolic hormones is usually perceived to be favourable, it has been realized that decreased action of GH, insulin, and IGF1 can increase life span and reduce tumour cell growth [1–3].

In vitro, insulin and IGF1 promote growth. Insulin was found to favour growth of breast cancer cells [4], and insulin increases proliferation of the MCF-7 breast cancer cell line [5–9], and IGF1 is a potent mitogen for osteosarcoma cells [8, 10–18]. These properties of insulin and IGF1 could underlie increased growth of breast cancer in obesity and of osteosarcoma in puberty. Insulin and IGF1 share mitogenic pathways, including the mitogen-activated protein kinase (MAPK), extracellular signal-regulated kinase (ERK) and the phosphatidylinositol 3-kinase (PI3K) pathways [8, 11]. Receptors for IGF1 (IGF1R) and insulin (IR) and their downstream signal transduction pathways thus became therapeutic targets [19].

Dietary restriction decreases circulating levels of insulin and IGF1, is antitumorigenic and increases life-span. The PI3K pathway may mediate the sensitivity of selected tumours to dietary restriction as tumours sensitive to dietary restriction were growth-responsive to insulin and IGF1 whereas tumours with constitutively active PI3K signalling were not. Decreased PI3K signalling in human tumour xenografts correlated better with dietary restriction-induced apoptosis than with decreased proliferation; enhanced apoptosis was shown to contribute to net tumour cell growth suppression [20].

We analysed in more detail antiapoptotic effects of insulin and IGF1 in two tumour cell lines, A549 non-small-cell lung cancer (NSCLC) and Saos-2/B10 osteoblastic osteosarcoma cells, with a special focus on the latter since they have been found to be critically dependent on insulin-like signals for proliferation and especially survival in vitro. Saos-2/B10 cells have been used to assess cancer-related safety of insulin analogues (IR binding agonists) with a focus on mitogenic potency, receptor activation and binding studies [13]. Increased affinity for the IGF1R was found to be strongly correlated with increased mitogenic potency as also confirmed by later studies [21]. Glargine, e.g., the first modified insulin to delay absorption and to prolong duration of action, has a 6–8-fold increased affinity for IGF1R and mitogenic potency compared with insulin [13, 21]. Nevertheless, glargine is considered as safe, because it is converted to metabolites M1 and M2 with decreased activities at both IGF1R and IR but an apparently more favourable ratio of metabolic–mitogenic potency than the native compound [22–25].

Overall, concentrations of insulin and insulin analogues in vivo (in healthy individuals and in patients with diabetes) are normally low compared to the levels required to elicit a mitogenic response. As indicated above, tumour growth could depend on both, regulation of apoptosis and mitogenesis. We therefore analysed in more detail antiapoptotic effects of insulin and IGF1 in the Saos-2/B10 cell line which was found to be critically dependent on insulin-like signals for proliferation and especially survival in vitro. We compared the effects of insulin and IGF1 to those of glargine and those of FCS, tested the interference of IGF-binding protein (IGFBP) 3 (inhibitor of IGF1) and of wortmannin (WT, inhibitor of PI3K), and analysed the signal transduction pathways involved.

## Materials and methods

### Cell culture

Saos-2 is an osteosarcoma cell line which was originally derived from a 14-year-old female (ATCC HTB 85, American Type Culture Collection. Rockville, MD); it was characterized in more detail and subcloned to yield the osteoblastic osteosarcoma line Saos-2/B10 [26, 27]. These cells are p53-negative [14, 28] and produce no IGF1 [10, 29]. They were passaged in Falcon tissue culture flasks in DMEM/Ham’s F-12 medium (Gibco 31885/21765) containing 50 µg/ml gentamicin, 2 mmol/l l-glutamine, and 10% (v/v) FCS at 37  °C in an atmosphere of 5% CO_2_. Cells (passage 16–33) were cultured in DMEM [50 µg/ml gentamicin, 2 mmol/l l-glutamine and 5% (v/v) FCS] for 3 days prior to the start of experiments. A549 is a lung (alveolar cell) adenocarcinoma cell line which was originally derived from a 58-year-old Caucasian male (ATCC, Rockville, MD; ECACC no. 86012804) [10, 30]. A549 cells belong to the family of NSCLC cells and to the adenocarcinoma histological subtype with epithelial origin. They contain wild-type p53 [31]. These cells were cultured (passage 7–24) in Falcon tissue culture flasks in RPMI (Gibco 31870) containing 2 mM l-glutamine, 50 µg/ml gentamicin, and 10% (v/v) FCS at 37 °C in an atmosphere of 5% CO_2_.

Under serum-free conditions, Ham’s F-12 medium was used with 1 g/l free fatty acid-free BSA (from Sigma, St. Louis, MO, USA). IGF1 was from Ciba-Geigy, Basel, Switzerland, insulin from Novo Nordisk, Gentofte, Denmark, and the insulin analogue glargine from Sanofi-Aventis, Frankfurt, Germany. rhIGFBP3 and pharmacological inhibitors of PI3K (WT: Sigma, Buchs, Switzerland) or MEK (UO126 or PD98059: Sigma, Buchs, Switzerland) were added as indicated.

### Assessment of proliferation

Cells (21 × 10^3^ per cm^2^) were plated in 5% FCS-containing medium. After 3 days of growth, confluent cultures were rinsed with serum-free medium, and incubated for 18 h in serum-free Ham’s F-12 medium containing 1 g/l BSA and growth factors as indicated. DNA synthesis was measured by methyl-[^3^H]-thymidine (Amersham, 89 Ci/mmol; 0.5 µCi per dish) incorporation during a 3-h pulse following exposure to test media for 18–21 h [32].

### Assessment of apoptosis

**Fig. 1:**
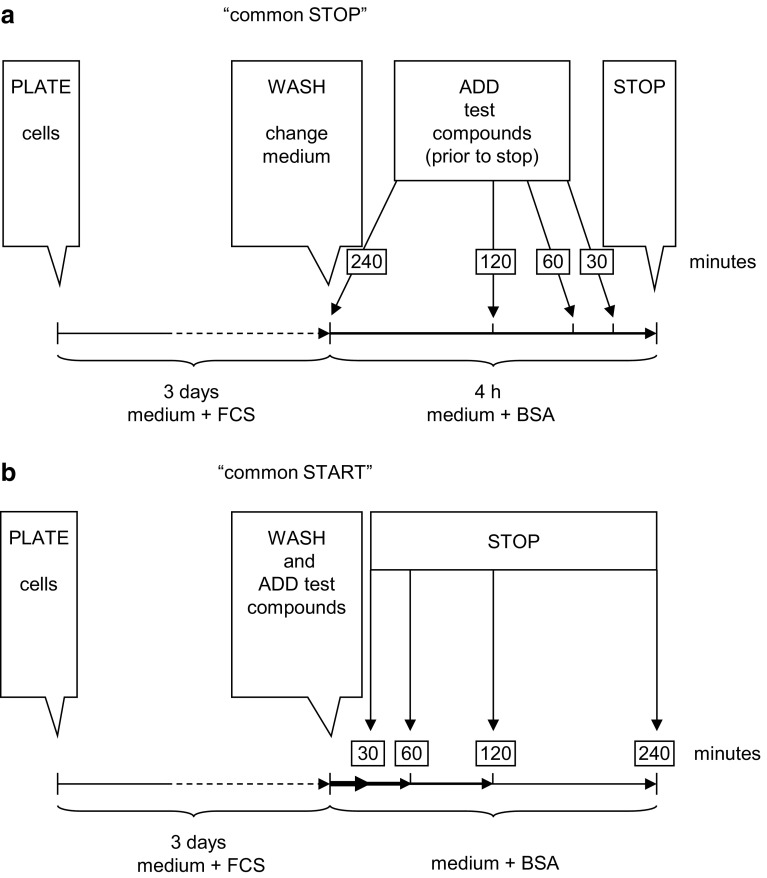
Flow diagram of experimental protocols. Cells were grown in FCS-containing media for 3 days and exposed to serum-free, albumin-containing test media as shown. Apoptosis was assessed after 4 h (**a**, “common stop”) by an ELISA detecting cytosolic oligonucleosomes. Insulin/IGF1 signalling was analysed by Western blotting using antibodies against the unphosphorylated and phosphorylated forms of IR-IGF1R-IRS1 (not shown), Akt/PKB and ERK1/2, and actin, with “common stop” (**a**) and with “common start” (**b**) protocols as indicated

Cells were plated as described for proliferation, washed, and incubated for 4 h in serum-free Ham’s F-12 medium containing 1 g/l BSA; growth factors and test compounds were added as shown in Fig. 1a.

Cells were washed with ice-cold PBS, and lysed into 400 µl of kit lysis buffer and centrifuged for 10 min at 200×*g* at room temperature. A 20 µl volume was used for analysis (Cell Death Detection ELISA^PLUS^ from Boehringer Mannheim) following the manufacturer’s instructions [29, 32]. This method measures cytosolic oligonucleosome-bound DNA and allows quantitative determination of histone-associated DNA fragments. It is based on a sandwich-enzyme immunoassay which detects enrichment of mono- and oligonucleosomes in the cytoplasmic fraction of cell lysates by monoclonal antibodies directed against DNA and histones [29].

### Immunoblotting

Cells were lysed in 50 mmol/l HEPES pH 7.5, 140 mmol/l NaCl, 1 mmol/l PMSF, 0.5% Triton X-100, 10 mmol/l NaF, 1 mmol/l Na_2_H_2_P_2_O_7_, 1 mmol/l Na_2_O_4_V, 3 µg/ml aprotinin, 3 µg/ml leupeptin. Equal amounts of protein (concentration determined using the bicinchoninic acid protein assay kit from Pierce) were separated by SDS-PAGE (NuPAGE, Invitrogen) and transferred onto nitrocellulose membranes. 2% Non-fat milk in TBST [10 mmol/l Tris–HCl, pH 7.4, 150 mmol/l NaCl, 0.05% (v/v) Tween 20] was used to block nonspecific binding of antibodies to membranes. Incubation with primary and secondary antibodies was either at room temperature for 1 h or overnight at 4 °C. Immuno-reactive proteins were visualized by the Lumi-Light Western blotting substrate (Roche) using a LAS-3000 imaging system (Fuji). Signal intensities were quantified using the AIDA software package (Raytest). Equal loading and transfer were confirmed with an antibody against actin (MAB1501: EMD Millipore, Temecula, USA). Insulin/IGF-dependent signalling was assessed with antibodies against the phosphorylated forms of Akt/protein kinase B (PKB; Ser473: Cell Signaling, Danvers, USA) and ERK1/2 (Thr202/Tyr204: Cell Signaling, Danvers, USA). Activation of IR and IGF1R was monitored using an antibody against p-Tyr (clone 4G10, EMD Millipore, Temecula, USA). Expression levels of Akt/PKB, ERK1/2, IR, IGF1R, IRS1, actin, and LC3A/B were assessed with specific antibodies [Akt/PKB: BD Transduction Laboratories, San Jose, USA; ERK1/2: Cell Signaling, Danvers, USA; insulin Rβ, IGF1Rβ, and IRS1: Santa Cruz, Dallas, USA; actin: clone C4, MAB1501, Millipore/Merck, Darmstadt, Germany; LC3A/B: (DRU4C) Rabbit mAb, Cell Signaling, Danvers, USA].

### Statistical analysis and presentation of data

At least three Western blots with duplicates or at least five ELISA or ^3^H-incorporation studies (carried out in triplicate), respectively, were combined. Data are expressed as means ± SEM. In the case of Western blots, results of quantifications are plotted on a log scale normalized to protein amount and relative to control; values were highly consistent between experiments regardless of normalization to protein (as loaded), total (phosphorylated plus nonphosphorylated proteins) Akt/PKB and ERK/MAPK or actin. Statistical significance was assessed by the unpaired two-sided Student’s *t*-test or by ANOVA. *p* < 0.05 was considered statistically significant.

## Results

### Insulin and IGF1 increase proliferation and potently inhibit apoptosis in Saos-2/B10 cells

**Fig. 2:**
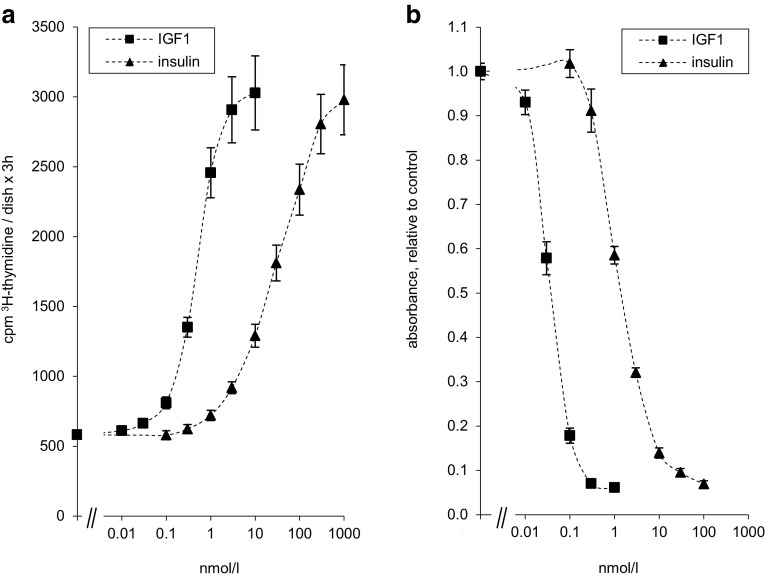
Insulin and IGF1 regulate proliferation and apoptosis of Saos-2/B10 cells. **a** Stimulation of [^3^H]-thymidine incorporation into DNA by IGF1 and insulin. Cells were grown in 5% FCS-containing media for 3 days and exposed to serum-free, albumin-containing test media, containing IGF1 or insulin. DNA synthesis was measured by a [^3^H]-thymidine pulse 18–21 h following exposure to test media (*n* = 6). **b** Inhibition of apoptosis by IGF1 and insulin. Cells were exposed to test media containing IGF1 or insulin as shown in Fig. 1a. Apoptosis was assessed after 4 h. Results are expressed relative to control (*n* = 5)

Insulin and IGF1 stimulated [^3^H]-thymidine incorporation into DNA in a dose-dependent manner, with increasing concentrations up to the same maximum (Fig. 2a). IGF1 was more potent and half-maximal stimulation was reached at lower concentrations of IGF1 (0.4 nmol/l) compared to insulin (20 nmol/l).

Serum withdrawal results in apoptosis of Saos-2/B10 cells within hours. Inclusion of IGF1 or IGF2 in test media protects Saos-2/B10 cells against apoptosis [29, 32]. We wanted to test if inclusion of insulin had a similar effect. Figure 2b shows dose–response curves for insulin and IGF1. Lower doses of IGF1 or insulin were sufficient to inhibit apoptosis compared to DNA synthesis. IGF1 was more potent compared to insulin with half-maximal values at 0.04 versus 1.0 nmol/l for insulin. IGF1 (at 1 nmol/l) and insulin (at 100 nmol/l) decreased apoptosis to a similar extent.

### Insulin and IGF1 activate Akt/PKB and ERK1/2 in Saos-2/B10 cells: time and dose dependence

**Fig. 3:**
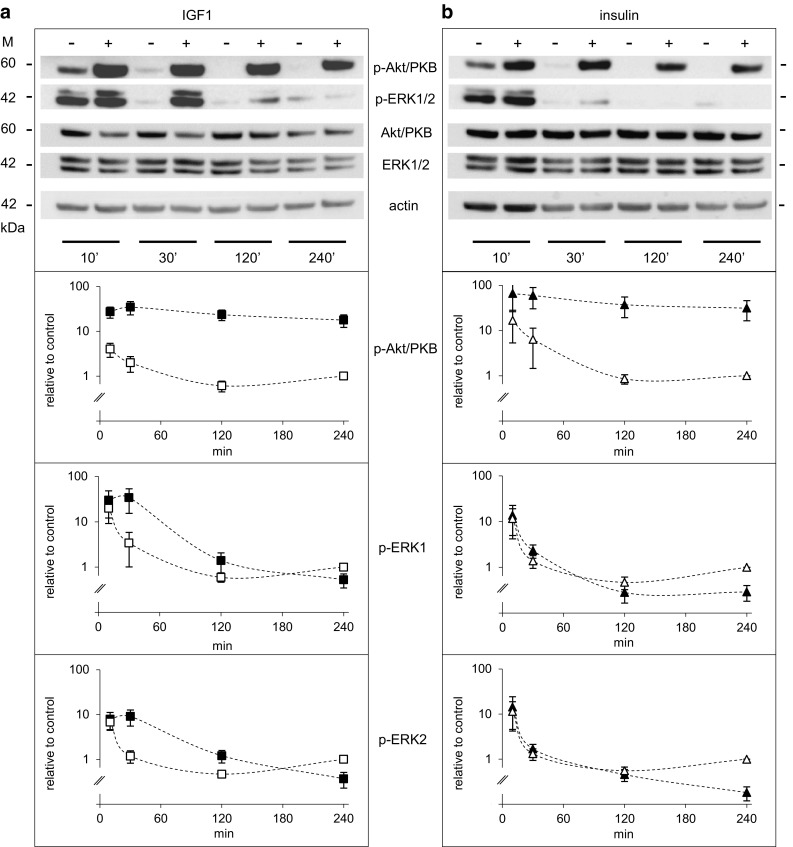
Time-dependent activation of Akt/PKB and ERK1/2 by IGF1 and insulin in Saos-2/B10 cells. Representative Western blots are shown on *top*; quantification of signals from at least four independent experiments below. Abundance of p-Akt/PKB and p-ERK1/2 is shown relative to control (4 h). Cells were exposed to vehicle (*empty symbols*), to 1 nmol/l IGF1 (*filled squares*) or to 100 nmol/l insulin (*filled triangles*) as outlined in Fig. 1b, and incubations were stopped after 10, 30, 120, and 240 min

**Fig. 4:**
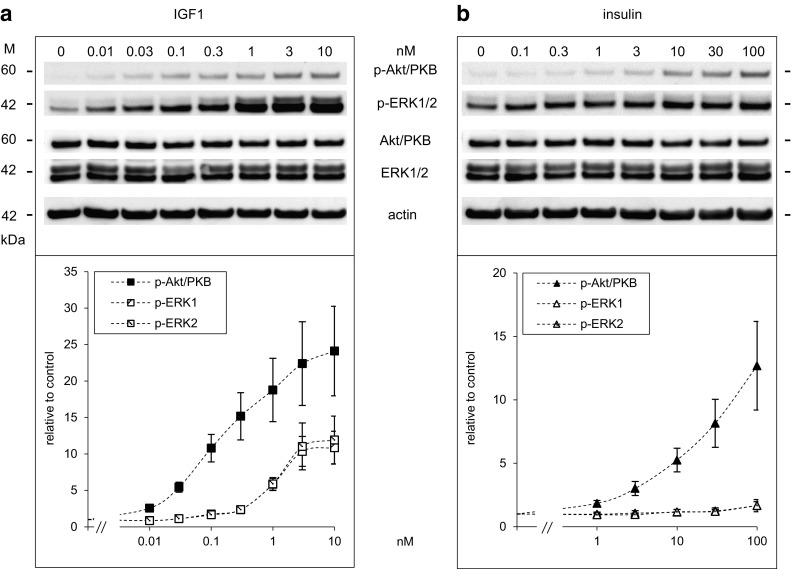
Dose-dependent activation of Akt/PKB and ERK1/2 by IGF1 and insulin in Saos-2/B10 cells. Cells were exposed to IGF1 (**a**) and insulin (**b**) for 30 min as described in Fig. 1b (common start). Representative Western blots are shown on *top*, below quantification of at least four independent experiments. Abundance of p-Akt/PKB and p-ERK1/2 is expressed relative to control

Activation of insulin/IGF1-dependent signalling in Saos-2/B10 cells was studied with phospho-specific antibodies against Akt/PKB (p-Ser473) and ERK1/2 (p-Thr202/Tyr204). Time dependence and dose dependence were analysed (Figs. 3, 4, respectively). IGF1 and insulin both activated Akt/PKB strongly within minutes; sustained activation of Akt/PKB was observed in response to both IGF1 and insulin while ERK1/2 activation was rather transient, and only observed in response to IGF1 (Fig. 3b). We consistently observed a decrease in phosphorylation of ERK1/2 after longer exposures (4 h), and insulin was more effective than IGF1 in this regard. Quantification of at least seven independent experiments is shown with numerical values (plotted on a log scale) in the lower panels.

Dose-dependent phosphorylation/activation of Akt/PKB and ERK1/2 after 30 min of exposure to IGF1 or insulin is shown in Fig. 4. IGF1 was more potent in phosphorylating Akt/PKB than insulin. The dose–response curves are broad, and our data cannot reveal whether maximal stimulation of p-Akt/PKB was achieved at 10 nmol/l IGF1; maximal activation was not reached at 100 nmol/l insulin, the highest concentration that was tested; thus, concentrations required for half-maximal stimulation of Akt/PKB (EC_50_) cannot be given. ERK1/2 phosphorylation dependent on stimulation with IGF1 was much less pronounced compared to Akt/PKB, and insulin even at high concentrations barely increased p-ERK1/2.

### Time-dependent inhibition of apoptosis in Saos-2/B10 cells by IGF1 and insulin

**Fig. 5:**
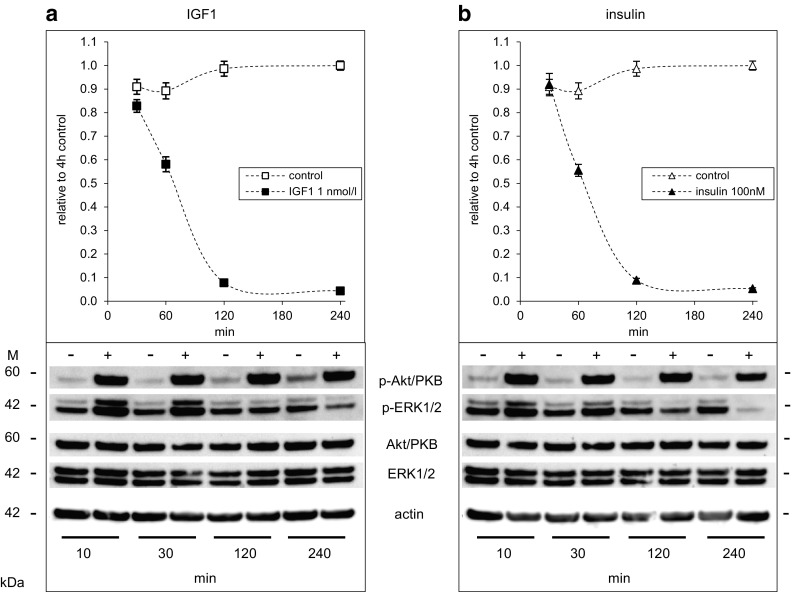
Time-dependent inhibition of apoptosis and activation of signalling by IGF1 and insulin in Saos-2/B10 cells. Cells were cultured in serum-free media for the last 4 h as shown in Fig. 1a (common stop). IGF1 (**a**, 1 nmol/l) or insulin (**b**, 100 nmol/l) was added to the media 30 min, 1 h, 2 h, or 4 h prior to stop. Apoptosis is shown at the *top* (expressed relative to 4 h control; *n* = 5), representative Western blots in *lower panels*

To test how long after serum withdrawal cells could be rescued from apoptosis, IGF1 or insulin were added to supernatants at different time points after serum withdrawal. Maximal inhibition of apoptosis was observed when IGF1 or insulin (or glargine, not shown) was added at least 2 h prior to the end of the 4-h incubation time (Fig. 5), i.e., not later than 2 h after serum withdrawal. At this time point, residual Akt/PKB phosphorylation in serum- and hormone-free control conditions was low (Fig. 3). IGF1 or insulin promptly increased Akt/PKB phosphorylation at all tested time points, i.e., also with the protocol of Fig. 1a, “common stop”, as well as with the protocol of Fig. 1b, “common start”. ERK1/2 was activated after short-term exposure to IGF1; strikingly, after 4-h exposure, p-ERK was consistently decreased in cells exposed to IGF1 or (more clearly) insulin, relative to control (Figs. 3, 5).

### IGFBP3 inhibits IGF1- but not insulin-dependent regulation of apoptosis in Saos-2/B10 cells

**Fig. 6:**
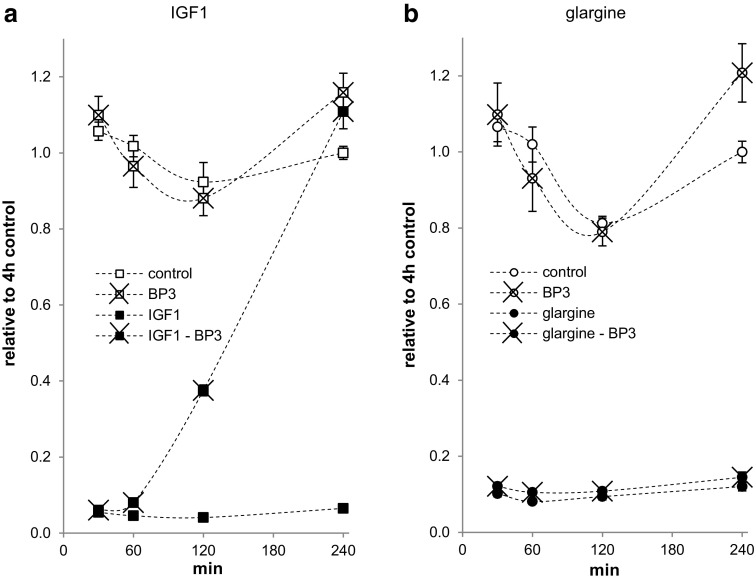
Time-dependent interference of IGFBP3 with apoptosis inhibition by IGF1 and glargine in Saos-2/B10 cells. Cells were cultured in serum-free media for 4 h in the absence or presence of 1 nmol/l IGF1 (**a**, *n* = 5) or 1 nmol/l glargine (**b**, *n* = 3) as shown in Fig. 1a (common stop). IGFBP3 (10 nmol/l) was added 30 min, 1 h, 2 h, and 4 h prior to stop (*n* = 5)

rhIGFBP3 can effectively block IGF1-induced proliferation and survival in cell culture experiments [17, 29, 32, 33]. In order to test whether apoptosis was regulated by IGF1 and insulin also in the presence of IGFBP3, cells were serum-deprived for 4 h in the absence or presence of IGF1, insulin, or glargine. 10 nmol/l rhIGFBP3 was added to the cells 30, 60, 120, or 240 min prior to the end of incubation (Fig. 6). Apoptosis was not changed in Saos-2/B10 cells exposed to rhIGFBP3 alone. 10 nmol/l rhIGFBP3 did not inhibit the apoptosis-preventing effects of 100 nmol/l insulin [32] and of 1 nmol/l glargine but selectively inhibited the apoptosis-preventing effects of 1 nmol/l IGF1; 10 nmol/l rhIGFBP3 was effective (in the presence of IGF1) if added at least 120 min prior to the end of the 4 h incubation time. Correspondingly, rhIGFBP3 blocked Akt/PKB phosphorylation dependent on incubation with 1 nmol/l IGF1 but not with 100 nmol/l insulin or 1 nmol/l glargine (not shown).

### Wortmannin inhibits IGF1- and insulin-dependent regulation of apoptosis in Saos-2/B10 cells

**Fig. 7:**
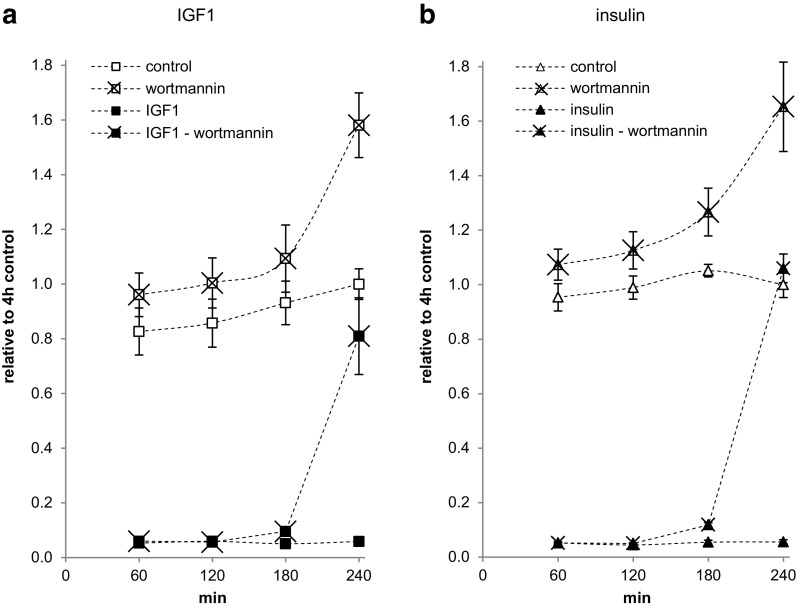
Time-dependent interference of wortmannin with apoptosis inhibition by IGF1 and insulin in Saos-2/B10 cells. Cells were cultured in serum-free media containing 1 nmol/l IGF1 (**a**, *n* = 5) or 100 nmol/l insulin (**b**, *n* = 3), as shown in Fig. 1a (common stop). WT (100 nmol/l) was added 1, 2, 3, and 4 h prior to stop

To test if activation of Akt/PKB and/or ERK1/2 are required for insulin/IGF1-dependent regulation of apoptosis in Saos-2/B10 cells, we used WT and UO126/PD98059 to inhibit their activation, respectively. We first confirmed by Western blotting that preincubating cells with these inhibitors for 10 min prior to exposure to 1 nmol/l IGF1 or 100 nmol/l insulin for 30 min blocked activation of Akt/PKB and/or ERK1/2. 100 (but not 20) nmol/l WT reliably blocked Akt/PKB phosphorylation, and 50 µmol/l UO126 or 20 µmol/l PD98059 blocked ERK phosphorylation under these conditions (not shown). WT (Fig. 7) but not UO126 or PD98059 (both not shown) interfered with protection from apoptosis by both IGF1 and insulin. WT had to be present for 4 h for this effect; however, more than half-maximal protection from apoptosis was still found when WT was added only for the last 3 h. In addition, cells cultured for 4 h in the presence of WT showed reduced activation of Akt/PKB by IGF1 and by insulin but marked phosphorylation of Akt/PKB was still observed when WT was added only for the last 3 h (not shown). WT alone, when present throughout 4 h, increased apoptosis in the control condition (Fig. 7).

### Comparing effects of IGF1 and insulin with those of glargine and FCS in Saos-2/B10 cells

**Fig. 8:**
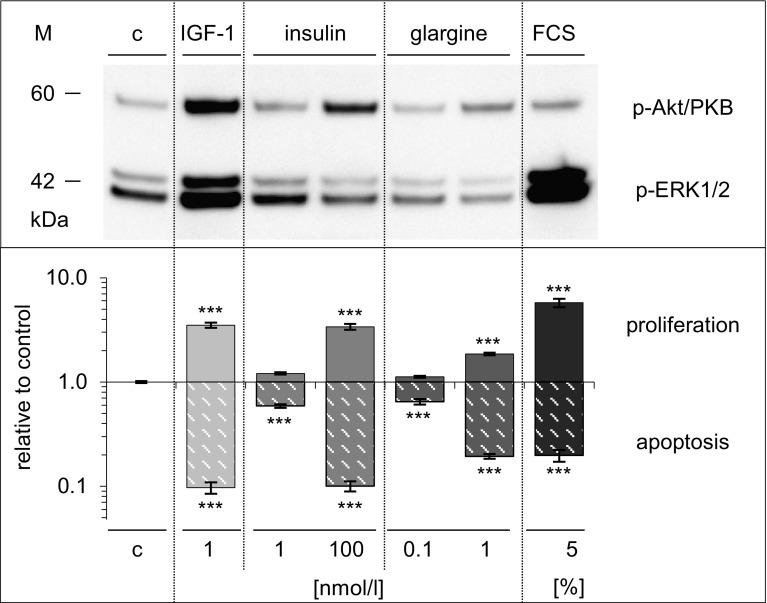
Effects of IGF1, insulin, glargine, and FCS compared in Saos-2/B10 cells. Cells were exposed to IGF1, insulin, glargine, or FCS as described for Figs. 2 and 4. *Top panel* Western blot showing p-Akt/PKB, p-ERK1/2, *bottom panel* stimulation of DNA synthesis and inhibition of apoptosis, expressed relative to control (*log scale*), *n* = 7. *c* denotes control, ***p < 0.0001

As in the case of IGF1 (low doses) and insulin (high doses), exposure of Saos-2/B10 cells to glargine also resulted in activation of Akt/PKB and protection from apoptosis (Fig. 8). Significant effects on apoptosis (but not on proliferation) were seen in response to 0.1 nmol/l glargine. Likewise, concerning activation of Akt/PKB and ERK1/2, it appeared that the potency of glargine was intermediate between that of IGF1 and insulin. FCS (5%) tended to be more effective in activating ERK1/2 and in stimulating DNA synthesis, but it tended to be less potent than IGF1 and insulin in protecting Saos-2/B10 cells from apoptosis and increasing p-Akt/PKB.

### Insulin and IGF1 regulate proliferation and apoptosis in A549 cells

In order to test if our findings in Saos-2/B10 can be reproduced in another human cell line, we repeated a subset of our experiments with A549 cells [10, 30].

**Fig. 9:**
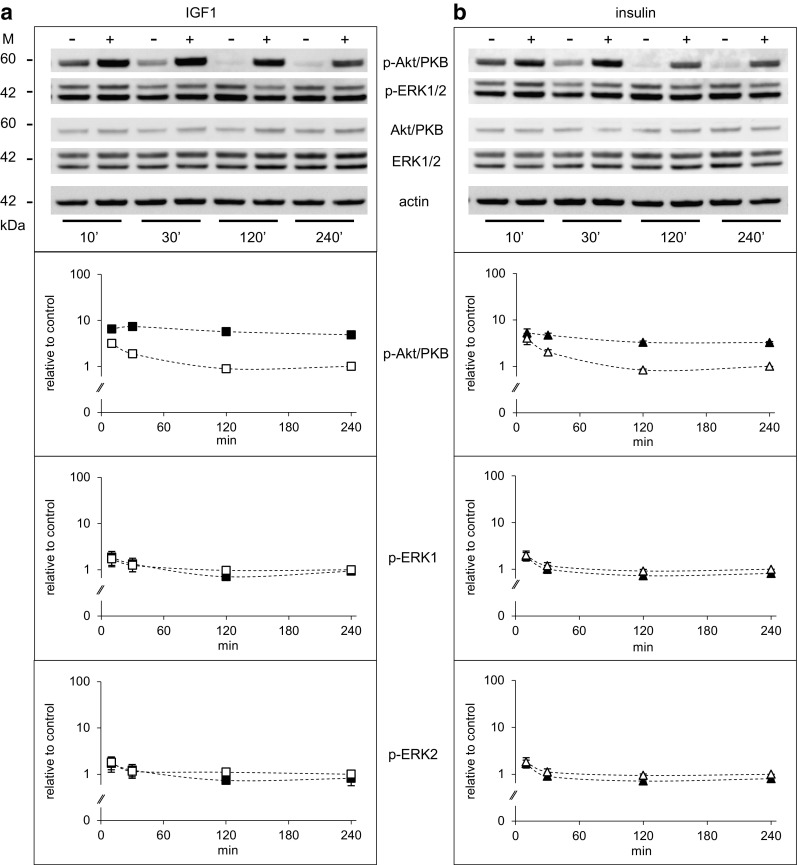
Time-dependent activation of Akt/PKB and ERK1/2 by IGF1 and insulin in A549 cells. Representative Western blots are shown on top; quantification of signals from two independent experiments (with duplicates) below. Abundance of p-Akt/PKB and p-ERK1/2 is shown relative to control (4 h). Cells were exposed to vehicle (*empty symbols*), to 1 nmol/l IGF1 (*filled squares*) or to 100 nmol/l insulin (*filled triangles*) as outlined in Fig. 1b, and incubations were stopped after 10, 30, 120, and 240 min (as in Fig. 3). *M* denotes markers

**Fig. 10:**
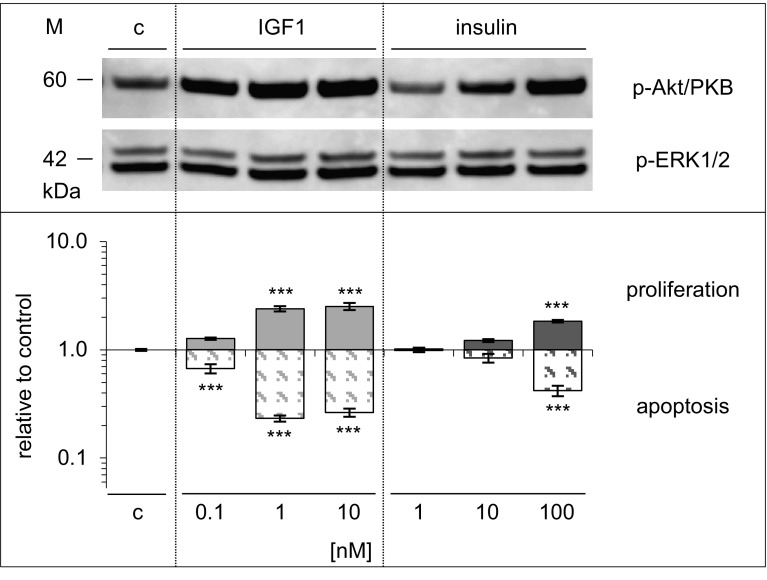
Dose-dependent effects of IGF1 and insulin on signalling, proliferation, and apoptosis in A549 cells. Cells were exposed to IGF1 or insulin as described for Figs. 2 and 4, and data are shown as in Fig. 8 for Saos-2/B10 cells. *Top panel* Western blot showing p-Akt/PKB, p-ERK1/2, *bottom panel* stimulation of DNA synthesis (*n* =  7 in triplicate) and inhibition of apoptosis (*n*  =  2 in triplicate), expressed relative to control (*log scale*). *c* denotes control, ***p  <  0.0001

First we analysed time- (Fig. 9) and dose-dependent (Fig. 10, top panel) regulation of Akt/PKB and ERK1/2 by insulin and IGF1 with phospho-specific antibodies against Akt/PKB (p-Ser473) and ERK1/2 (p-Thr202/Tyr204). IGF1 and insulin both activated Akt/PKB strongly within minutes in A549 cells as they did in Saos-2/B10 cells; sustained activation of Akt/PKB was observed in response to both IGF1 and insulin. ERK1/2 activation was less pronounced and transient.

Figure 10 summarizes dose-dependent activation of Akt/PKB and ERK1/2 as well as regulation of proliferation and apoptosis in A549 cells. Insulin or IGFI both protected A549 cells from apoptosis and stimulated DNA synthesis. As in Saos-2/B10 cells, IGF1 (at lower doses) and insulin (at higher doses) were more effective in increasing and maintaining Akt/PKB phosphorylation and protecting cells from apoptosis while regulation of ERK1/2 and stimulation of DNA synthesis was less sensitive.

We also tested the effects of IGFBP3 and of WT in A549 cells (not shown). For both test compounds, results were as found for Saos-2/B10 cells. rhIGFBP3 blocked the effects of IGF1 but not those of insulin; per se, it slightly enhanced apoptosis which appears consistent with a role for endogenous IGF1 in the survival of A549 cells; autocrine IGF1 may also be related to their lower IGF1R number and their lower sensitivity to insulin/IGF1 as compared with Saos-2/B10 cells [10]. WT fully blocked IGF1- and insulin-stimulated Akt/PKB phosphorylation, and it decreased basal/residual p-Akt/PKB within 30 min of exposure. WT also interfered with protection from apoptosis by both 1 nmol/l IGF1 and 100 nmol/l insulin, and, when present throughout 4 h, increased apoptosis in the control condition.

## Discussion

Osteosarcoma is a malignant bone tumour with an incidence peak coinciding with the adolescent growth spurt, a time when the hormonal milieu is characterized by high GH, IGF1 and insulin levels. Osteosarcoma growth may be particularly dependent on IGF1 [12, 16, 18]. The Saos-2/B10 subline model has been well characterized and used extensively to elucidate actions of IGF1, IGF2, IGFBPs, insulin, and analogues [10, 13, 17, 29, 32, 33].

IGF1 and insulin activated Akt/PKB in Saos-2/B10 cells and concomitantly protected against apoptosis (EC_50_ for inhibition of apoptosis, 0.04 nmol/l for IGF1, 1 nmol/l for insulin). Saos-2/B10 cells were (approximately 10-fold) more sensitive to IGF1 in terms of inhibition of apoptosis than with regard to stimulation of DNA synthesis. In the case of insulin (and of glargine), this difference was even more pronounced, approximately 20-fold. Apoptosis was inhibited to a similar minimum by IGF1, insulin, or glargine, but higher concentrations of insulin or glargine were required. Effects of IGF1 but not those of insulin and glargine were blocked by IGFBP3. FCS-containing media (without addition of IGF1 or insulin) also activated Akt/PKB and protected from apoptosis (Fig. 8). Media containing 5% FCS were more effective than IGF1 or insulin in activating ERK1/2/MAPK and in stimulating DNA synthesis, but less potent than 1 nmol/l IGF1 or 100 nmol/l insulin in protecting Saos-2/B10 cells from apoptosis within 4 h, and 5% FCS was also less effective in stimulating p-Akt/PKB within 30 min (Fig. 8). These findings together with the blocking effects of WT are in line with the notion that signalling through IGF1R/IR and Akt/PKB promotes survival of Saos-2/B10 cells upon serum withdrawal. Most previous studies assessed DNA synthesis in vitro and suggested that insulin concentrations that stimulated DNA synthesis in vitro were probably not reached in vivo [13, 34]. However, as we show here, this is not necessarily the case when considering antiapoptotic effects. Insulin (either endogenous or exogenous) could well reach concentrations which may contribute to survival of selected malignant tumour cells, especially in insulin-resistant patients. In view of the low concentrations required for apoptosis prevention, the potential of insulin and analogues in maintaining certain malignant cells in a vital state may have been underestimated. It appears that characterization of IR binding agonists should include assays on prevention of apoptosis and not restrict the focus on mitogenic potency. It has been proposed that specificity of ligand–receptor interactions defines biological response. Usually, insulin promotes proliferation of tumour cells only at higher concentrations than IGF1, possibly since it predominantly acts via type 1 IGFRs. Glargine is more potent than insulin with regard to IGF1R phosphorylation [35–37] and with regard to stimulation of proliferation [7, 9, 13, 21, 35]. Glargine is also (around seven to eight times) more potent than insulin with regard to inhibition of apoptosis. However, insulin and IGF1 have overlapping receptor binding characteristics and share intracellular signalling pathways, including IR substrates, PI3K- and ERK-dependent pathways [38]; specificity of insulin versus IGF action is far from being understood [39].

An important finding of our study is that IGF1 and insulin effectively increase and maintain Akt/PKB in its phosphorylated state and concomitantly protect the cells from undergoing apoptosis. Inhibition of apoptosis was sensitive to WT, a widely used inhibitor of the PI3K-Akt/PKB axis [40–42]. IGF1 and insulin induced a prompt, strong and sustained activation of Akt/PKB while ERK1/2 was activated only transiently, and only by IGF1 and not by insulin. Compounds preventing activation of ERK1/2 (UO126 and PD98059) failed to inhibit the apoptosis-preventing effects of IGF1 and insulin. Our observation that insulin effectively prevented apoptosis yet barely activated ERK1/2 also suggests that prevention of apoptosis by insulin or IGF1 does not depend on activation of ERK1/2. In this context, it appears less important at which receptor(s) signalling initiates, as signals finally converge at the level of PI3K to prevent apoptosis. Therefore, we propose that in vitro safety tests with insulin analogues should include assays addressing apoptosis and activation/maintenance of PI3K-dependent signalling, rather than activation of different IR/IGF1R types. Indeed, Akt/PKB is central to a well-studied survival pathway and a known protooncogene [43–46]. Akt/PKB is frequently hyperactivated in osteosarcoma [19]. It promotes cell survival by phosphorylating and inactivating several pro-apoptotic targets, such as Bad and the forkhead transcription factors. Overall, Akt/PKB is one of the most frequently activated kinases in human cancer, and ectopic expression of a dominant-negative kinase-dead variant inhibited the ability of IGF1 to prevent apoptosis in Saos-2 cells [11]. Our findings with Saos-2/B10 cells concur with the report by Kalaany and Sabatini [20] who also came to the conclusion that continuous exposure to insulin-like signals possibly more often helps tumour cells to survive than directly favours tumour growth progression by increasing mitogenesis.

Insulin-regulated autophagy was reported recently to link diabetes and cancer [47]. Indeed, autophagy may induce or prevent cell death, dependent on the cells under study and the context, as also reported for osteosarcoma cells [48]. Microtubule-associated protein 1 light chain 3 (MAP-LC3) is converted from the LC3-I (16 kDa) to the active LC3-II (14 kDa) form indicating increased autophagy flux. In our cell culture conditions, LC3-II did not appear to increase after serum withdrawal, and decreased only to a minor extent by IGF1 and insulin after 2 and 4 h (supplemental Fig. 1). Within the short time frame of our experimental setting, we found no obvious correlation between phosphorylation of Akt/PKB and induction of autophagy, in contrast to the strong correlation between phosphorylation of Akt/PKB and inhibition of apoptosis. Our study does not allow to clarify or exclude a potential role of autophagy in the regulation of apoptosis.

We show experiments just for two cell lines, and results therefore should be confirmed in other models. In both cell lines, we observed the same close relationship between activation and maintenance of Akt/PKB and protection from apoptosis. However, there were quantitative differences regarding potencies of IR/IGF1R agonists which may be due to known variations in expression levels of IGF1 and IGF1R [10]. It has to be kept in mind that Saos-2/B10 cells are particularly sensitive to IR/IGF1R agonists; other tumour cell types could be less sensitive. Another limitation of our study was the use of pharmacological inhibitors to downregulate the activation of Akt/PKB and ERK1/2. Such compounds could exert unspecific effects, especially with longer incubation times. However, we have focused our study on the regulation of apoptosis in the short term (up to 4 h) where fewer overall toxic effects are expected. Conclusive evidence for the requirement of Akt/PKB in insulin/IGF1-induced survival of Saos-2/B10 cells requires specific knock-down (e.g., by siRNAs). To test if their activation is not only required for insulin/IGF1 effects but also sufficient for regulation of survival ectopic activation of downstream components (as, e.g., in [49]) would be required.

In healthy humans, insulin is secreted in pulsatile bursts [50]. Not only loss of early phase insulin response to glucose but also the disruption of high frequency pattern of insulin secretion during fasting is characteristic of beta cell failure in type 2 diabetes [51]. Indeed, insulin given as intravenous pulses has greater glucoregulatory activity than insulin by continuous infusion [52, 53]; this may account for increased insulin dose requirement of flat profile long-acting insulin analogues [54]. Normal regulation of blood glucose in healthy subjects with pulsatile insulin secretion includes periods of low insulin concentrations. In contrast, standard basal insulin replacement modalities fail to mimic this physiological insulin secretion pattern. Our in vitro study addressed insulin/IGF-dependent regulation of apoptosis and phosphorylation of Akt/PKB under conditions of continuous or interrupted stimulation. Within the 4 h following FCS withdrawal IGF1 and insulin effectively activated Akt/PKB and prevented apoptosis, also when added with a 2-h delay (Fig. 5). Moreover, p-Akt/PKB was decreased and protection from apoptosis was lost when IGF1 was blocked by later addition of IGFBP3 for at least 2 h (Fig. 6).

Our results indicate that apoptosis is prevented in Saos-2/B10 and A549 cells by signalling through the PI3K-Akt/PKB pathway which is activated and remains active upon continuous exposure to IGF1 or insulin. Furthermore, lower concentrations of IR/IGF1R agonists are required for preventing cell death than for stimulating cell proliferation.

## Electronic supplementary material

Below is the link to the electronic supplementary material.

Supplementary material 1 (PPTX 873 KB)

Supplementary material 2 (DOCX 10 KB)
