# Supplementary material for: Prevention of tumour cell apoptosis associated with sustained protein kinase B phosphorylation is more sensitive to regulation by insulin signalling than stimulation of proliferation and extracellular signal-regulated kinase
Source: Mol Cell Biochem. 2017 Mar 18;432(1):41–54. doi: 10.1007/s11010-017-2996-y (PMC5532423; doi:10.1007/s11010-017-2996-y)
Supplement: Supplementary file 1 — Supplementary material 1 (PPTX 873 KB) [file 11010_2017_2996_MOESM1_ESM.pptx]

## Slide 1
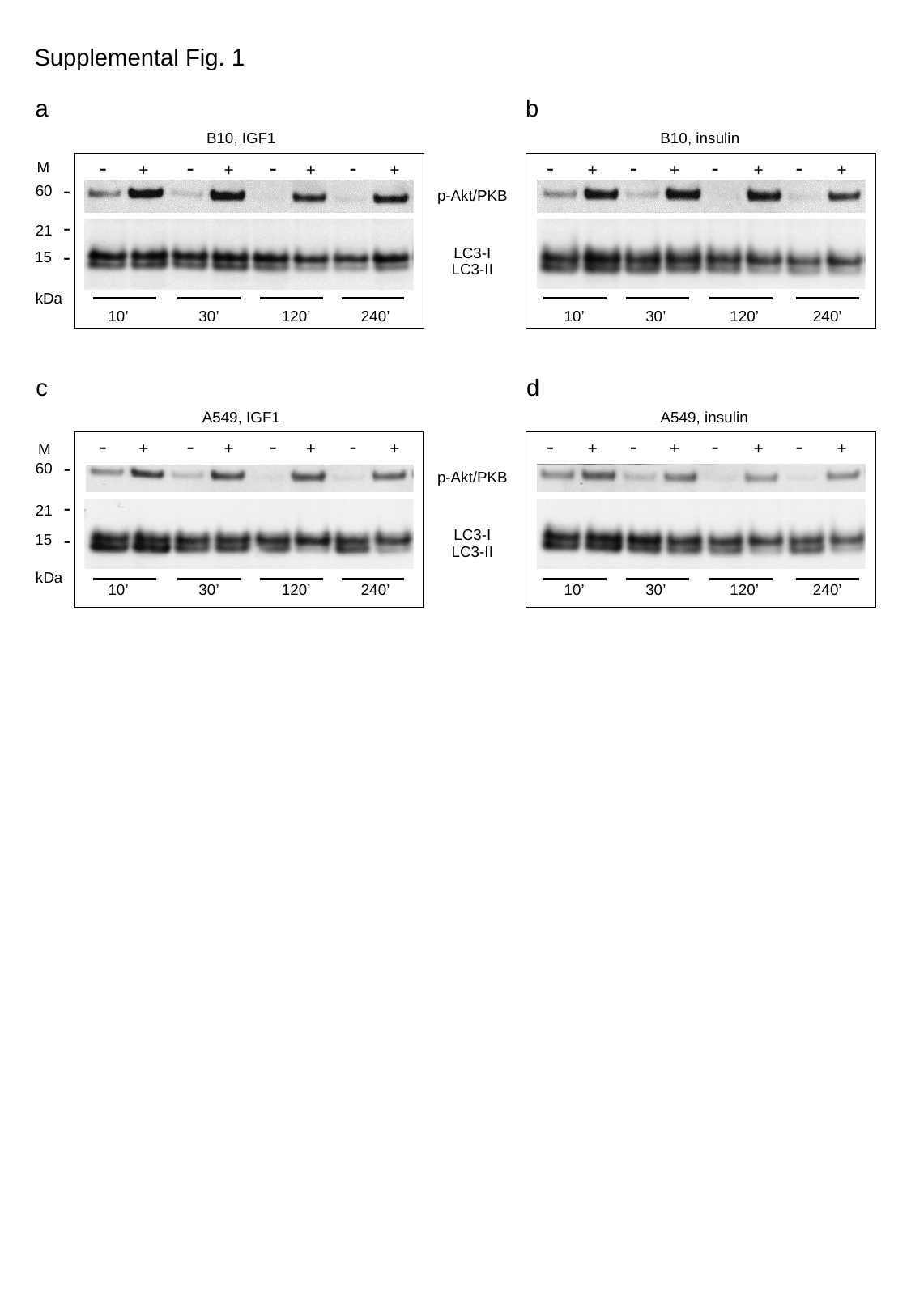

Supplemental Fig. 1
a
b
B10, IGF1
B10, insulin
-
-
-
-
-
-
-
-
M
+
+
+
+
+
+
+
+
-
60
p-Akt/PKB
-
21
LC3-I
-
15
LC3-II
kDa
10’
30’
120’
240’
10’
30’
120’
240’
c
d
A549, IGF1
A549, insulin
-
-
-
-
-
-
-
-
+
+
+
+
+
+
+
+
M
-
60
p-Akt/PKB
-
21
LC3-I
-
15
LC3-II
kDa
10’
30’
120’
240’
10’
30’
120’
240’
