# Supplementary material for: Prevention of tumour cell apoptosis associated with sustained protein kinase B phosphorylation is more sensitive to regulation by insulin signalling than stimulation of proliferation and extracellular signal-regulated kinase
Source: Mol Cell Biochem. 2017 Mar 18;432(1):41–54. doi: 10.1007/s11010-017-2996-y (PMC5532423; doi:10.1007/s11010-017-2996-y)
Supplement: Supplementary file 2 — Supplementary material 2 (DOCX 10 KB) [file 11010_2017_2996_MOESM2_ESM.docx]

**Supplemental Fig.1** Time-dependent regulation of p-Akt/PKB and LC3-I/LC3-II by IGF1 and insulin in Saos-2/B10 and in A549 cells. Western blots show experiments performed as shown in Figs. 3, and 9, i.e. cells were exposed to vehicle, to 1 nmol/l IGF1 (left panels) or to 100 nmol/l insulin (right panels) as outlined in Fig. 1b, and incubations were stopped after 10, 30, 120, and 240 min. Blots were probed with antibodies against the phosphorylated form of Akt/PKB (Ser473) to detect p-Akt/PKB and with antibodies against LC3. Upper panels (a and b), Saos-2/B10 cells, lower panels (c and d), A549 cells. M denotes markers
